# Association Between Polymorphisms of *DRD2*, *COMT*, *DBH*, and *MAO-A* Genes and Migraine Susceptibility: A Meta-Analysis

**DOI:** 10.1097/MD.0000000000002012

**Published:** 2015-10-30

**Authors:** Hu Chen, Chun-Xue Ji, Lian-Li Zhao, Xiang-Jun Kong, Xian-Tao Zeng

**Affiliations:** From Department of Scientific Research, Cangzhou Central Hospital, Cangzhou, Hebei Province, China (HC); Department of Neurology, Cangzhou Central Hospital, Cangzhou, Hebei Province, China (C-XJ, HC); Department of Human Resources, Cangzhou Central Hospital, Cangzhou, Hebei Province, China (L-LZ); Central Laboratory, Cangzhou Central Hospital, Cangzhou, Hebei Province, China (X-JK); and Center for Evidence-Based and Translational Medicine, Zhongnan Hospital of Wuhan University, Wuhan, China (X-TZ).

## Abstract

Some epidemiological studies have investigated the relationship between genetic polymorphisms of *DRD2*, *COMT*, *DBH*, and *MAO-A* and migraine susceptibility, but the results are still inconsistent. Thus, our aim was to further assess the association through a meta-analysis.

We examined 5 single nucleotide polymorphisms (SNPs) in 4 genes, including *DRD2* rs1799732 and rs6275, *DBH* rs7239728, *MAI-A*-VNTR, and *COMT* rs4680, and performed a meta-analysis of 11 published case–control studies including 3138 cases and 4126 controls. Odd ratios (ORs) with 95% confidence intervals (95% CIs) were used to evaluate the association between the 5 genetic polymorphisms and migraine susceptibility.

There was no significant relationship between migraine susceptibility and 4 genetic polymorphisms of *DRD2* rs1799732 and rs6275, *DBH* rs7239728, and *MAO-A*-VNTR. Nevertheless, decreased risk of migraine was observed to be in association with *COMT* rs4680 polymorphism in overall analysis (AA vs. GG + GA: OR = 0.76, 95% CI = 0.60–0.97, *P*_Het_ > 0.642, I^2^ = 0), and in Caucasian group after subgroup analysis (AA vs. GG + GA: OR = 0.75, 95% CI = 0.58–0.96, *P*_Het_ > 0.433, I^2^ = 0).

Studied polymorphisms of *DRD2*, *DBH*, and *MAO-A* genes may not be associated with migraine susceptibility. However, *COMT* rs4680 polymorphism may decrease the risk of migraine, especially in Caucasians. The failure to evaluate environmental influence and provide adjusted effect size estimates highlights the need for additional studies in a large number to take these factors into consideration, thus better elucidating the role of the genes tested in migraine.

## Abstract 2

Supplemental Digital Content is available in the text

## INTRODUCTION

Migraine, the most common neurological disease, seriously threatens the health of people all over the world, and has some symptoms including headache, nausea, vomiting, fatigue, irritability, and nervousness. Patients with migraine are generally aged from 25 to 50, and the risk of migraine in females is 3 times higher than that in males.^1–3^ Genetic factors have been found to be involved in the etiology of the disease.^4–7^

Dopamine, a hormone and neurotransmitter of catecholamine and phenethylamine, plays an important role in the repair of nervous system in human brain and associates with decreased dopamine activities. Several diseases in nervous system are associated with dysfunctions of dopamine system, including migraine and Parkinson disease. Dopamine receptor D2 (*DRD2*) gene locates on chromosome 11q22.2–22.3, and its most studied single nucleotide polymorphisms (SNPs) include rs1799732 and rs6275. The former, a deletion polymorphism (−141C Ins/Del), correlates with reduced DRD2 expression, and the latter is a synonymous polymorphism locating in exon 7 of *DRD2* gene.

Isoenzymes monoamine oxidases A (MAO), involved in catabolism of monoamine neurotransmitters, catalyzes oxidative deamination and participates in functional regulation of cell structures.^8,9^*MAO* gene can be divided into 2 subtypes of *MAO-A* and *MAO-B* according to different distributions and autoimmune features of cells.^10,11^*MAO-A* gene locates on chromosome Xpll.23, and has a high affinity to endogenous neurotransmitters, and one of its most studied SNP, VNTR, contains a 30 bp long repeated sequence. Catechol-*O*-methyltransferase (COMT) is an enzyme inactivating catecholamines. The *COMT* gene functional polymorphism rs4680 can affect the enzyme activities.^12^*DBH* gene, with 12 exons and a length of 23 kb, locates on chromosome 9q34.^13^ The SNP rs7239728 in the promoter region of *DBH* gene is associated with phenotypic variations in plasma.

Genetic factors have been implicated in enzyme activities, and they, to some extent, can result in DNA damage, and finally cause the occurrence of diseases. Several studies have investigated the relationship between genetic polymorphisms of *DRD2*, *COMT*, *DBH*, and *MAO-A* genes and migraine susceptibility.^14–24^ But the results are conflicting rather than conclusive. Our meta-analysis combining 3138 cases and 4126 controls aims to provide a more precise estimation of the association. Pooled odds ratio (OR) was the main outcome of this meta-analysis.

## MATERIALS AND METHODS

### Search Strategy and Inclusion Criteria

We searched Pubmed, CNKI, and Embase for relevant studies using the combination of the items “*DRD2*” or “*COMT*” or “*DBH*” or “M*AO-A*,” “polymorphism,” and “migraine.” All eligible studies evaluating the association between polymorphisms of the 4 genes and migraine susceptibility were selected according to the following criteria: with a case–control design; stating sufficient data for calculating pooled ORs with 95% confidence intervals (95% CIs). Studies were precluded if they were case-only studies, duplicates or with unrelated titles and abstracts. As all analyses were performed based on previous published researches, the ethical approval and patient consent are not required.

### Data Extraction

Two investigators independently extracted requisite data from all eligible studies according to the identical criteria. The extracted data included: the name of first author, publication year, ethnicity, country of origin, numbers of cases and controls, genotyping methods, genotype frequencies, and *P*-value for Hardy–Weinberg equilibrium (HWE) in control group. Inconsistent data were discussed between the 2 investigators until reaching a consensus.

### Quality Assessment

Assessment of the methodological quality of observational studies was done independently by 2 investigators. A risk-of-bias score modified from a previous meta-analysis^25^ was used (Table S1, http://links.lww.com/MD/A513). The score has 4 domains: information bias: ascertainment of cases and controls, assessment of genotyping assay, confounding bias: population stratification and common confounding variables were evaluated, selective reporting of outcomes, HWE was assessed among controls. The full score was 21. Studies awarded at least 14 scores were defined as low-bias studies.

### Statistical Analysis

Pooled ORs with 95% CIs were utilized to evaluate the relationship between polymorphisms of the 4 genes and migraine susceptibility. Heterogeneity among included studies was detected by Q test and I2 metric. Pooled ORs were calculated with a fixed-effects model when *P*-value >0.05 and I^2^ < 50%, which indicated low possibility of heterogeneity; otherwise, a random-effects model was used. Publication bias was examined by Begg funnel plot and Egger test. HWE was checked in control groups by χ^2^ test. Sensitivity analysis was conducted, by removing the independent studies (one at a time) and reestimating the pooled ORs, to test the stability and robustness of the combined estimates. Statistical analysis was performed using STATA version 12.0 (Stata Corporation, College Station, TX).

## RESULTS

### Study Characteristics

**FIGURE 1:**
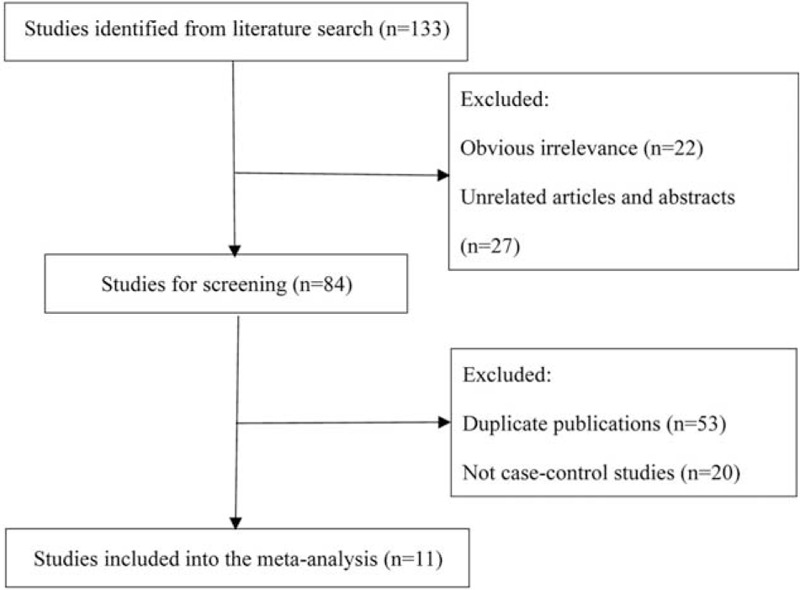
Flow diagram of the study selection process for the meta-analysis.

As displayed in Figure 1, a total of 133 articles were identified from databases, and 122 of them were precluded for duplicates, unrelated titles and abstracts, case-only studies, and obvious irrelevance. Finally, 11 papers were included into our meta-analysis.^14,17,21,23,24,26–31^ All these studies had a low risk of bias, with the total score ranging from 14 to 17. The main characteristics of included studies are displayed in Table 1.

**TABLE 1:**
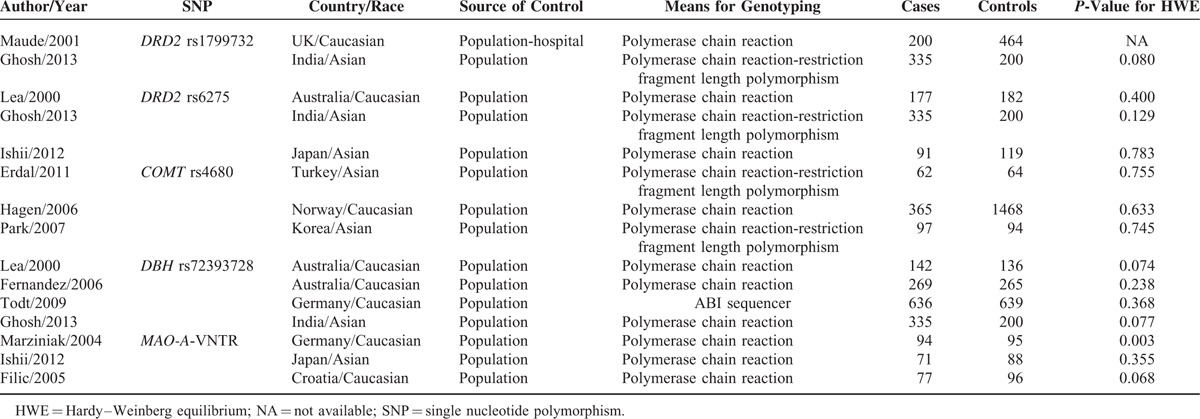
Principal Characteristics of the Studies Included in the Meta-Analysis

### Meta-Analysis

Lack of an association between polymorphisms in *DRD2*, *DBH*, *MAO-A* and migraine

As shown in Table 2, DRD2 rs1799732 was not associated with migraine risk when assuming a homozygote (OR = 11.55, 95% CI = 0.67–199.78), dominant (OR = 1.18, 95% CI = 0.79–1.77), recessive (OR = 11.35, 95% CI = 0.66–196.14), allele (OR = 1.05, 95% CI = 0.91–1.23), and heterozygote model (OR = 1.09, 95% CI = 0.72–1.64), without significant heterogeneity (*P*_Het_ > 0.05, I^2^ < 50%). Analysis of *DRD2* rs6275 provided a fixed-effect OR of 0.94 (95% CI = 0.67–1.32) in homozygote model, of 1.01 (95% CI = 0.83–1.22) in dominant model, of 0.91 (95% CI = 0.67–1.24) in recessive model, of 0.98 (95% CI = 0.84–1.15) in allele model and of 1.03 (95% CI = 0.82–1.28) in heterozygote model, with low possibility of heterogeneity (*P*_Het_ > 0.05, I^2^ < 50%). Stratified analysis by ethnicity indicated no association signals either in Asians or Caucasians (Table 2). A meta-analysis of *DBH* rs7239728 in all samples showed no sign of association with migraine (homozygote: OR = 1.02, 95% CI = 0.86–1.22; dominant: OR = 0.98, 95% CI = 0.88–1.11; recessive: OR = 0.98, 95% CI = 0.88–1.11; allele: OR = 1.02, 95% CI = 0.93–1.12; heterozygote: OR = 0.95, 95% CI = 0.82–1.10), with no significant heterogeneity (*P*_Het_ > 0.05, I^2^ < 50%). No novel association was observed in subgroups (Table 2). In a meta-analysis of *MAO-A*-VNTR, no association signals were shown at genotype and allele levels (homozygote: OR = 1.01, 95% CI = 0.69–1.48; dominant: OR = 1.06, 95% CI = 0.82–1.38; recessive: OR = 0.80, 95% CI = 0.57–1.13; allele: OR = 0.97, 95% CI = 0.79–1.19; heterozygote: OR = 1.18, 95% CI = 0.84–1.66). No obvious heterogeneity was seen within these studies (*P*_Het_ > 0.05, I^2^ < 50%). Subgroup analysis did not show any relationship in Caucasians and Asians (Table 2).

**TABLE 2:**
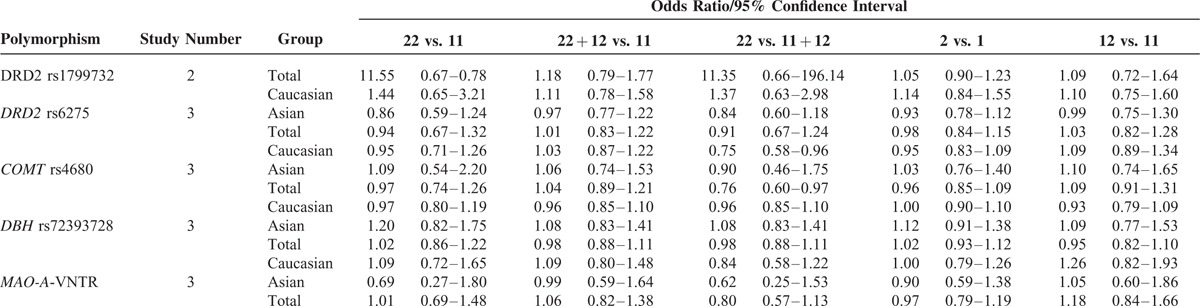
Polymorphisms of *DRD2*, *COMT*, *DBH*, and *MAO-A* Genes and Migraine Risk

### Decreased Migraine Risk and *COMT* rs4680

**FIGURE 2:**
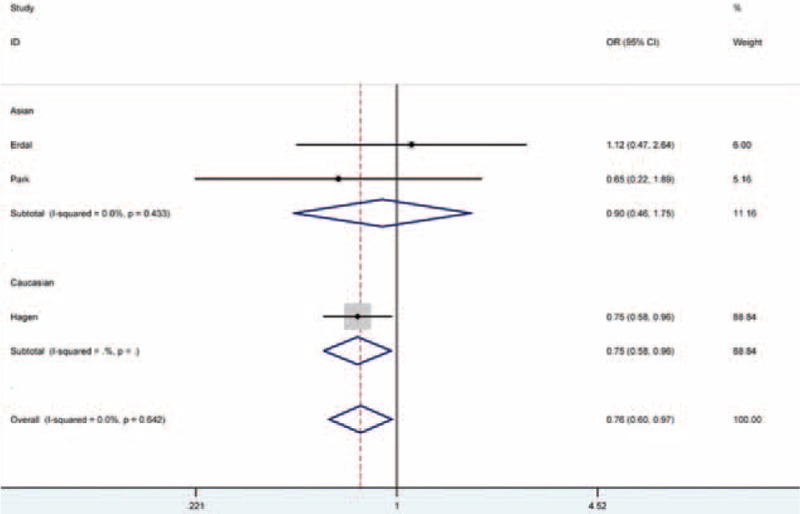
Forest plot of migraine susceptibility associated with *COMT* rs4680 polymorphism under AA versus GG + GA genetic model.

In a meta-analysis of all studies concerning *COMT* rs4680 and risk of migraine, carriers of the AA genotype versus carriers of GG + GA genotypes had 24% decreased migraine risk (AA vs. GG + GA: OR = 0.76, 95% CI = 0.60–0.97, *P*_Het_ > 0.642, I^2^ = 0). In subgroup analysis, the AA genotype versus the GG + GA genotypes was 0.75-fold less likely to develop the disease in Caucasian populations (AA vs. GG + GA: OR = 0.75, 95% CI = 0.58–0.96, *P*_Het_ > 0.433, I^2^ = 0), as displayed in Figure 2. No signals of relationship were seen in other genetic models tested (Table 2).

### Sensitivity Analysis

Sensitivity analysis was conducted by excluding one single study at a time to observe alterations in whole results which had no substantial difference before and after the deletions, suggesting our meta-analysis results were stable and credible.

### Publication Bias

**FIGURE 3:**
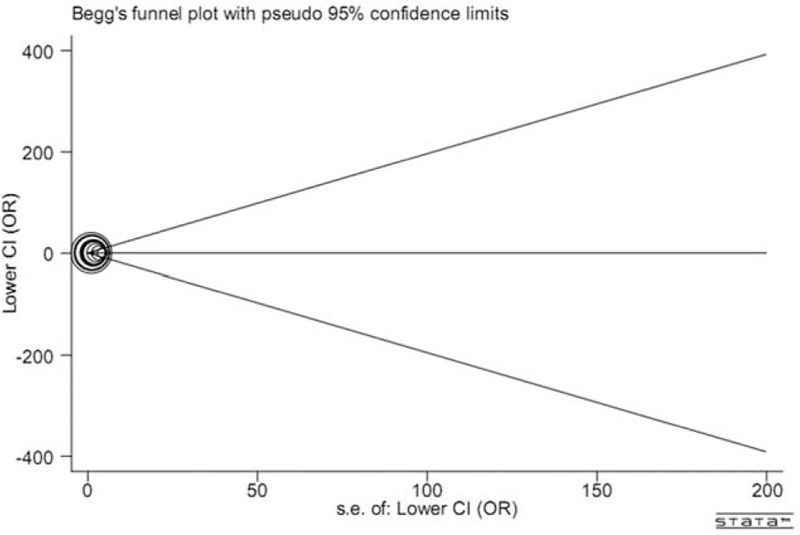
Begg funnel plot of publication bias.

The shape of the funnel plot seemed symmetrical (Fig. 3), implying negligible publication bias. Additionally, Egger test provided further statistical evidence for the absence of significant bias (*P* = 0.748).

## DISCUSSION

Although the pathophysiology of migraine remains incompletely understood, an effect conferred by dopamine was implicated almost 40 years ago.^32^ Such influence is further illustrated by an observation that dopamine shows signs of hypersensitivity in patients with migraine and that dopamine receptors can mediate nociception, autonomic responses, and vascular tone.^33^ Dopamine antagonists have reportedly been used to eliminate migraine-related symptoms and headache.^33^ Evidence from a murine model suggests that dopamine receptors usually appear in the trigeminovascular pathway and that dopamine is able to inhibit nociceptive trigeminovascular transmission.^34^ These reports highlight the central role of dopamine in migraine and make the dopamine-related genes such as *DRD2*, *COMT*, *DBH*, and *MAO-A* candidates.

Accumulating evidence supports a relationship between these genes and migraine development. A 7-transmembrane receptor protein of the dopamine pathway predominantly expressed in pars compacta of the substantia and neostriatum is encoded by the *DRD2*.^35^ Genetic polymorphisms in this locus are known to have important functional consequences. Expression-based research in vitro revealed 2 times higher expression levels of *DRD2* in individuals with Ins/Ins of rs1799732 than those with Del/Del.^36^ Multiple studies in human models have also connected rs6275 to DRD2 transcript instability and reduced translational efficiency.^37^ Several other polymorphisms in the gene, such as rs7131056 can directly affect the function of DRD2 product.^28^ For rs7239728, a promoter polymorphism in *DBH*, has been linked to phenotypic variability in DBH activity in plasma.^38^ Migraineurs versus controls have been reported to have significantly lower plasma norepinephrine levels.^39^ The DBH catalyzes the dopamine-to-norepinephrine conversion, and reduced DBH activity in individuals with rs7239728 might be a cause of lower norepinephrine in migraneurs.^15^ The *VNTR* polymorphism in the MAO-A gene is associated with higher enzyme expression of MAO-A.^40^ The functional impact leads to increased MAO-A activity, a cause of hypermetabolism of amine neurotransmitters and decreased levels of serotonin, conditions previously implicated in migraine pathophysiology.^41^ All these results point to the high possibility of a relationship between *DRD2*, *DBH*, *MAO-A* and risk of migraine.

Multiple epidemiological studies have investigated the association between migraine susceptibility and polymorphisms of the four genes *DRD2*, *COMT*, *DBH*, and *MAO-A*. The study by Ghosh et al^15^ demonstrated no significant association between *DRD2* rs6275 polymorphism and susceptibility to migraine. In another large-scale study, the researchers provided opposite results by showing an increased risk of migraine associated with *DRD2* rs6275 and 1799732 polymorphisms.^14^ Fernandez et al^27^ found no significant relationship between *DBH* rs72393728 polymorphism and migraine susceptibility in their research. For the *MAO-A*-VNTR polymorphism, Ishii et al.^30^ identified an increased risk of migraine. In addition, the study in Finns by Tammimaki and Mannisto^42^ demonstrated some evidence for an increase in the migraine risk associated with *COMT* rs4680 polymorphism.

In our meta-analysis, there was no significant association between migraine susceptibility and 4 SNPs in 3 genes, including *DRD2* rs1799732 and rs6275, *DBH* rs7239728, and *MAO-A*-VNTR. However, *COMT* rs4680 polymorphism was associated with a decreased risk of migraine, especially in Caucasians. There are abundant data supporting these findings. The rs4680 polymorphism results in a transition of valine (Val) to methionine (Met) at codon 158, and it is the amino acid substitution causes decreased thermostability and enzymatic activity and increased dopamine-degrading activity.^43–45^ While the findings for *DRD2* rs1799732 and rs6275, *DBH* rs7239728, and *MAO-A*-VNTR are inconsistent with the results from previous functional studies previously introduced in this section, the results identified for *COMT* rs4680 are in accordance with the published reports.

Due to some limitations, the present findings should be explained prudently. First, we demonstrated evidence for the absence of association for *DRD2* rs1799732, *DRD2* rs6275, *DBH* rs7239728, *MAO-A*-VNTR and the presence of association for *COMT* rs4680. These results may be caused by the limited data available for each polymorphism. Hence, we cannot exclude the probability that the association for the former 4 polymorphisms will be significant and that the association for the latter would be lost after the enlargement of the sample size. Second, we put equal emphasis on English and non-English publications during literature search. However, only those papers written in English were identified. In addition, no unpublished data were included. Thus, selection bias may have occurred, though there was no indication of significant bias in Begg funnel plot and Egger test. Third, we merely assessed the genetic effects on migraine risk, not considering environmental influence. Fourth, the results were based on unadjusted data, which might affect the accuracy of the results. Finally, subgroup analyses based on age, gender, and other potential confounding variables were not performed because of insufficient data.

In conclusion, our meta-analysis demonstrates a significant association between decreased risk of migraine and *COMT* rs4680 polymorphism, but no association for *DRD2* rs1799732, *DRD2* rs6275, *DBH* rs7239728, and *MAO-A*-VNTR. Large-scale studies where gene-environment interactions are considered and adjusted effect is estimated are needed to determine the role of these dopamine-related genes in migraine, thus providing new insights into the mechanisms that underlie the disease pathogenesis.
